# Antimetastatic activity of novel ruthenium (III) pyridine complexes

**DOI:** 10.1002/cam4.826

**Published:** 2016-09-07

**Authors:** Liwei Gu, Xiaodong Li, Qingsen Ran, Chen Kang, Canghai Lee, Jianying Shen

**Affiliations:** ^1^ Institute of Chinese Materia MedicaChina Academy of Chinese Medical SciencesBeijing100700China

**Keywords:** Invasion, metastasis, RuCl_4_‐pyridine complexes, ruthenium, tumor

## Abstract

Ruthenium‐based complexes have emerged as promising anticancer, especially antimetastatic agents. Among them, NAMI‐A (*trans*‐[Ru(III)Cl_4_ (DMSO)(Im)][ImH], Im = imidazole, DMSO = dimethyl sulfoxide) was well studied. In this study, we studied the antimetastatic activities of two novel NAMI‐A derivatives containing pyridine, G26b and G94a, using cultured cells and tumor‐bearing mice. Same to NAMI‐A, these two complexes displayed little direct cytotoxicity to the cancer cells in vitro and in vivo*,* but they, especially G26b, significantly reduced the occurrence and development of lung metastases in mice bearing the 4T1 mammary carcinoma. In vitro, these two complexes displayed significant suppressive effect on invasion and migration of cells and tube formation of human umbilical vein endothelial cell, to the same extent of NAMI‐A. The transcription of important molecules involved in metastasis, matrix metalloproteinase 2 and 9 (MMP‐2 and ‐9), and vascular endothelial growth factor, was suppressed by the two complexes, as well as NAMI‐A. Plasma atomic emission spectrometer showed G26b had a longer Ru‐elimination time in lung, which may be a reason for better antimetastatic effect of G26b than NAMI‐A. Our results have demonstrated that G26b is a more effective antimetastatic agent than NAMI‐A.

## Introduction

Cancer is the leading killer of human being all over the world. More than 90% of cancer death results from metastasis 1, which consists of many steps through which cancer cells disseminate from the primary site, propagate, and form secondary cancers at distant sites 2. The nuclear role of metastasis and invasion, therefore, represents an important therapeutic target. As a group of zinc‐dependent endopeptidases, matrix metalloproteinases (MMPs), was found collectively able to degrade all the components of the extracellular matrix (ECM), and thus, correlated with the late stages of tumor invasion and metastasis 3. It has been reported that up‐regulation of MMPs expression contributes to invasion and metastasis of cancer cells 4, 5, 6. Among the human MMPs reported to date, MMP‐2 and ‐9 are the key enzymes involved in degrading Type‐I and ‐IV collagens and ECM 7, 8. They contribute to cancer invasion, metastasis 5, 9, and angiogenesis 10. Unfortunately, until now, in spite of substantial research, the metastasis remains a serious clinical problem and its mechanisms are still incompletely understood 11.

Therefore, to develop agents with effective antimetastatic activity, but with low host cytotoxicity is important in anticancer research. Currently, inorganic drugs such as cisplatin and its related compounds are successfully used in the treatment of many cancer types with high incidence 12, 13. NAMI‐A (*trans*‐[Ru(III)Cl_4_(DMSO)(Im)][ImH], Im = imidazole, DMSO = dimethyl sulfoxide) was the first ruthenium anticancer agent ever to reach the stage of clinical trials 4, 14. It has been demonstrated that NIMA‐A is more effective and selective against metastatic cancer cells, with no appreciable toxicity against liver, kidney, or lung 15, 16. Nevertheless, many research groups have been persistently explored—new ruthenium complexes with more potential in antitumor activity 12, 17, 18, 19, 20.

**Figure 1:**
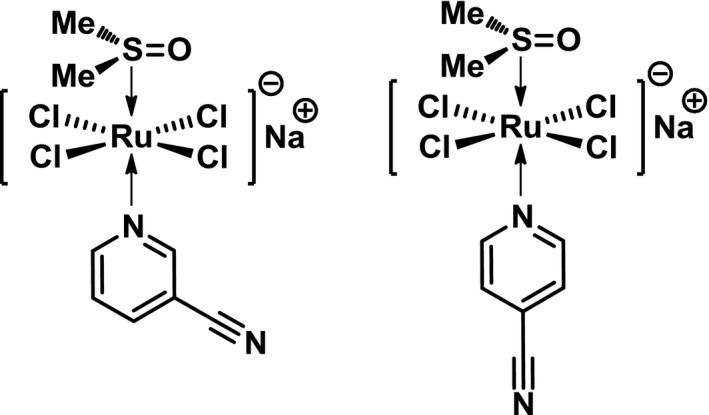
The chemical structures of G26b (left) and G94a (right).

Two novel NAMI‐A derivatives containing pyridine: G26b and G94a (*trans*‐ [Ru(III)Cl4(DMSO)(CNpy)]Na.2DMSO,CNpy = 3‐cyanopyridine, G26b; CNpy = 4‐cyanopyridine,G94a) (Fig. 1) have been designed and synthesized in our institute recently. In this study, using cultured cancer cells in vitro and tumor‐bearing mice in vivo, we studied their anticancer activities. It was found that these two complexes had more effective antimetastatic effect than NAMI‐A. They significantly reduced the occurrence and development of lung metastases in mice bearing the 4T1 mammary carcinoma. In vitro, these two NAMI‐A pyridine derivatives also displayed significant suppressive effect on invasion and migration. Our study has demonstrated that these novel complexes, especially G26b, are more promising than NAMI‐A as antimetastatic agents.

## Materials and methods

### Materials

NAMI‐A, and its two derivatives, G26b and G94a (*trans*‐ [Ru(III)Cl4(DMSO)(CNpy)]Na.2DMSO, CNpy = 3‐cyanopyridine, G26b; CNpy = 4‐cyanopyridine,G94a) were supplied by Prof. Guogang Liang research group in the Institute of Chinese Materia Medica, China Academy of Chinese Medical Sciences, who designed and synthesized the two novel complexes. The structures are shown in Figure 1.

The MTT [3‐(4, 5‐dimethylthiazol‐2‐yl)‐2, 5‐diphenyltetrazoliumbromide]‐based colorimetric assay kit was purchased from Roche (Indianapolis, IN). RPMI 1640, DMEM, fetal bovine serum (FBS), and sodium pyruvate were purchased from Gibco BRL (Grand Island, NY). Gemcitabine was from Lilly (Fegersheim, France). The standard substance of ruthenium was purchased from National Center of Analysis and Testing for Nonferrous Metals and Electronic Materials (NCATN, Beijing, China).

### Cell lines and animals

Human mammary carcinoma MCF‐7, mouse mammary carcinoma 4T1, human lung cancer A549, mouse lung cancer Lewis (LLC), and human umbilical vein endothelial cells (HUVEC) were obtained from the Institute of Basic Medical Sciences of Chinese Academy of Medical Sciences (Beijing, China).

BALB/c female mice (aged 4–6 weeks) were purchased from the Academy of Military Medical Science (Beijing, China). Animal experiments were conducted according to the institutional ethical guidelines for animal experiments and approved by the Institutional Animal Care and Use Committee (IACUC) at Institute of Chinese Materia Medica, China Academy of Chinese Medical Sciences.

### In vitro experiments

#### Cell culture and treatments

A549, 4T1, and HUVEC cell lines were maintained in RPMI 1640 culture medium (Gibco, Gaithersburg, MD), MCF‐7 and LLC were maintained in DMEM medium. The medium was supplemented with 10% FBS (PAA, Pasching, Austria) and 100 units/mL penicillin G, 100 units/mL streptomycin, and 1 mM NaHCO_3_. All cell lines were kept at 37°C, 5% CO_2_ humidified incubator.

#### Measurement of cell viability

Cells were plated in 96‐well plates and cell viability was determined by MTT reduction assay. After incubation, the cells were treated with MTT solution (final concentration, 5 mg/mL) for 4 h. The dark blue formazan crystals formed in intact cells were solubilized with DMSO and absorbance at 490 nm was measured with a microplate reader (Rayto, Shenzhen, China).

#### Transwell migration assay

Migration assays were done in Transwell tissue culture plates (6.5 mm and 8 *μ*m pore size, Corning Incorporated). 4T1 cells were kept for 24 h in a serum‐free –medium, then they were trypsinized and resuspended in RPMI‐1640 medium with 2% FBS, and placed in the upper chamber of the Transwell insert (2 × 10^4^ cells/well) and incubated with ruthenium (III) complexes and RPMI‐1640 medium containing 10% FBS was added to the lower chamber. The cells were then fixed with methanol and stained with crystal violet. The migrated cells in five random 100× fields were counted. Each experiment was done in triplicates, and the experiment was repeated twice.

#### In vitro wound‐healing assay

HUVEC cells were seeded in 6‐well plates and grown overnight to confluence. The monolayer cells were scratched with 10 *μ*L pipette tip to create a wound. The cells were washed twice with PBS to remove floating cells and then incubated in medium without serum. The rate of wound closure was investigated by photography 24 h later. Each value was derived from three randomly selected fields (40×).

#### Neovascularization formation assays

The 96‐well plate was coated with 50 *μ*L Matrigel (BD Biosciences, San Jose, CA, USA). A total of 2 × 10^4^ HUVEC cells were suspended in 100 *μ*L and treated with media or 20 *μ*mol/L of G26b, G94a, and NAMI‐A incomplete medium for 5 h. The network formation was observed under an inverted phase contrast microscope (Olympus, Tokyo, Japan). Total tubes and tube branches formed were measured and compared from three different viewing fields at 40× magnification. Tube formation assays were performed in triplicates and two independent experiments were conducted.

#### RNA preparation and real‐time PCR

Quantitative PCR was performed using SYBR Green real‐time PCR method. Total RNA was isolated with an RNA extraction kit (TIANGEN BIOTECH, Beijing, China), and the concentration of total RNA was measured spectrophotometrically (Nano Drop 8000; Thermo, Hudson, New Hampshire, USA). Quantitative RT‐PCR was performed on an ABI 7500 PCR instrument (Applied Biosystems, Foster City, CA) using two‐stage program parameters provided by the manufacturer as follows: 2 min at 50°C, 10 min at 95°C, and then 40 cycles of 95°C for 15 sec, 60°C for 32 sec. Each sample was tested in triplicates using quantitative RT‐PCR, and samples obtained from three independent experiments were used for the analysis of relative gene expression data using the 2^−ΔΔCT^ method 21.The primers used in this study were listed as follows: VEGF (Forward: AAAACACAGACTCGCGTTGC; Reverse: ATCTGGTTCCCGAAACCCTG), MMP‐2 (Forward: ACAGCAGGTCTCAGCCTCAT, Reverse: TGAAGCCAAGCGGTCTAAGT), MMP‐9 (Forward: TGCGCTACCACCTCGAACTT; Reverse: GATGCCATTCACGTCGTCCT), and GAPDH (Forward: GGCTGAGAACGGGAAGCTTGTCAT; Reverse: CAGCCTTCTCCATGGTGGTGAAGA). The quantity of each transcript was calculated as described in the instrument manual and normalized to the amount of GAPDH, a housekeeping gene.

### In vivo experiments

#### Xenografts

The mouse 4T1 mammary carcinoma cells 5 × 10^5^ were injected subcutaneously into female BALB/c mice on D0. Mice bearing cancers were randomized into five groups (*n* = 10) on D5. Experimental groups were administrated intraperitoneally (i.p.) with G26b, G94a, or NAMI‐A at a dose of 17.5 mg/kg per day for consecutive 6 days, and then administrated for three times at 1‐day interval. Vehicle control group was administrated with normal saline and positive group was administrated i.p. with gemcitabine at a dose of 50 mg/kg once every 5 days for three times. Mouse weight was measured twice per week. Mice were killed on D21.

The same dosage regimen was designed to observe the survival time of mice‐bearing cancer. The mice were then randomized into three groups (*n* = 11): Vehicle, NAMI‐A, and G26b. The survival end point was set as either spontaneous death of the animal or killed on D40.

#### Biodistribution of RuCl4‐cynpy complexes

The collected lungs were weighed and then dissolved in digest solutions (65% HNO_3_), such that the amount of acid added was equal to ten times the weight of the tissue sample. The samples were heated by step‐heating method at 120–180°C for 40 min. After cooling down to room temperature, each of resulting solutions was then diluted by deionized water to 5 mL, and subsequently analyzed by plasma atomic emission spectrometer ICPE‐9000 (Shimadzu, Japan) to determine the total amount of Ru in each measured organ. A calibration plot was developed for Ru at the concentration range of 0–320 *μ*g/mL. Three animals per group were used in the biodistribution measurement. The concentration of Ru in each sample was determined by emission intensity at 240.272 nm.

### Statistical analyses

Results were subjected to computer‐assisted statistical analysis using ANOVA and Dunnett's *t‐*test. Differences of *P *<* *0.05 were considered to be significantly different from the controls.

## Results

### Cytotoxic effects on different cell lines

**Figure 2:**
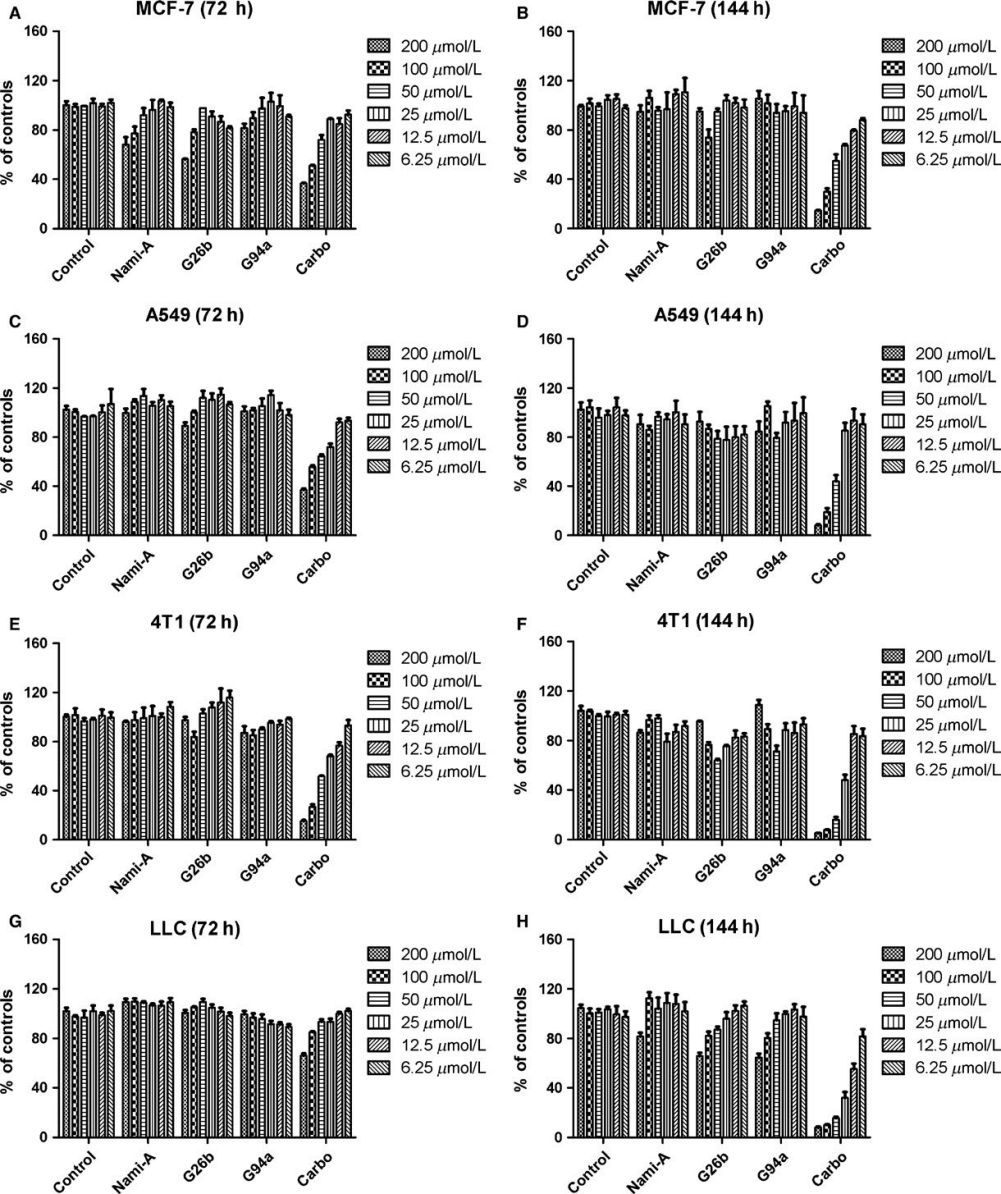
Dose–response histograms of the cytotoxic effects of the tested ruthenium‐pyridine complexes in tumor cell lines. The cytotoxicity of ruthenium‐pyridine complexes to four different cell lines were measured after 72 and 144 h treatment. Different from carboplatin, G26b, G94a, and NAMI‐A displayed no significant cytotoxicity against cancer cell lines. Data represent the means of triplicate samples ±SD.

The cytotoxicity of ruthenium‐pyridine complexes to four different cell lines were measured after 72 and 144 h treatment, and the results are summarized in Figure 2. It was apparent that G26b and G94a were little cytotoxic in vivo. Most of the IC_50_ values could not be determined because the values were out of the concentration range used in the experiments and would be higher than 200 *μ*mol/L. Different from carboplatin, NAMI‐A and the two ruthenium complexes displayed no significant cytotoxicity against cancer cell lines. The results were consistent to the reported findings 22.

### Suppression of breast cancer growth and metastasis in mice

**Figure 3:**
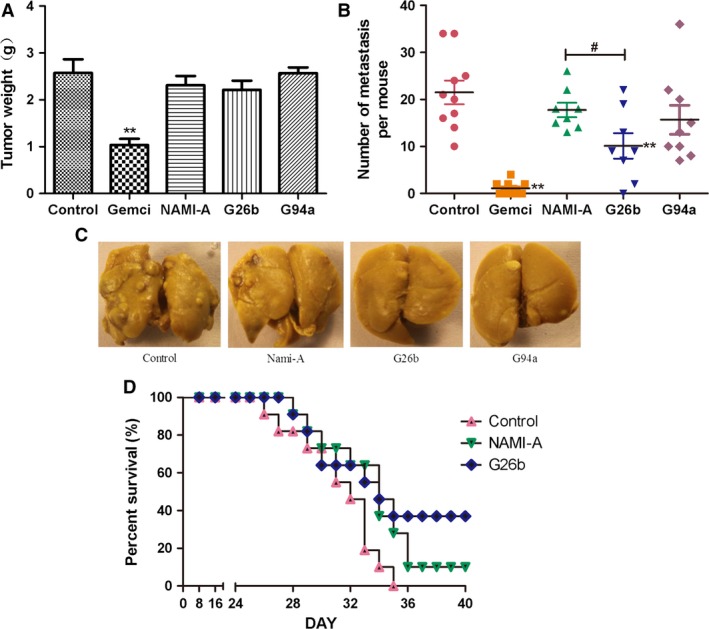
The inhibition of tumor growth on mammary carcinoma 4T1 xenograft. (A) Tumor mass 21 days after tumor cell inoculation (*n* = 10). (B) Metastasis development 21 days after tumor inoculation. The graph shows the number of lung metastasis tumors (*n* = 8). (C) Examples of metastatic nodules in the lungs of mice in B. (D) Survival curve of mice (*n* = 11). All experiments were performed three times with similar results. **P <* *0.01 compared with the control group. G26b versus NAMI‐A group at the same dose, ^#^stands for *P *<* *0.05.

To detect the anticancer activity of the complexes in vivo, we treated disease model mice of breast cancer 4T1 with G26b, G94a, and the reference compound NAMI‐A. The 5‐day initiation period of primary cancer was followed by therapeutic intervention, intraperitoneal administration of tested complexes at the dosage of 17.5 mg/kg per day. Both the primary cancer growth and cancer metastasis in lungs were measured after treatment. Although the primary cancer growth was not suppressed by the treatment, cancer metastasis was suppressed significantly by RuCl4‐cynpy complexes, especially G26b (Fig. 3A–C). No retinal toxicity or hepatotoxicity was found in mice treated with the tested complexes (data not shown).

Because G26b displayed significantly stronger antimetastatic effect than NAMI‐A, we compared the survival time of mice treated with these two complexes. The survival curve is shown in Figure 3D. Longer lifetime was observed in the G26b group.

### Effect of G26b and G94a on cell migration and invasion

**Figure 4:**
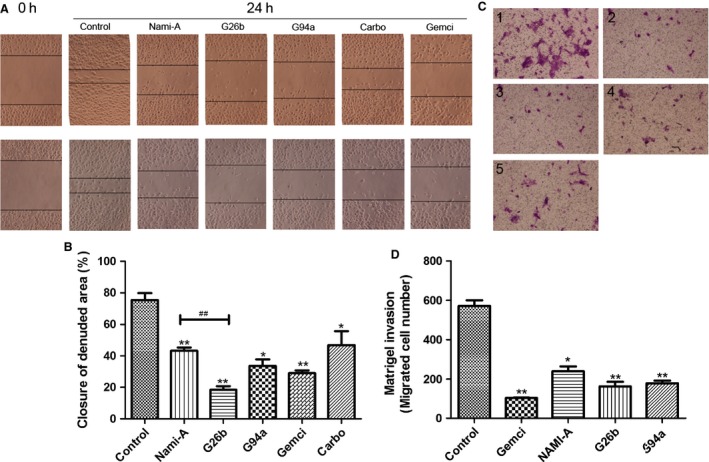
Inhibitory effects of ruthenium‐pyridine complexes on migration and invasion. (A–B) Effects of G26b on the migration in human umbilical vein endothelial cells. Cells were scratched with a pipette tip and then treated with gemcitabine (1 *μ*g/mL) and G26b, G94a, NAMI‐A, and Carboplatin (20 *μ*mol/L) and for 24 h. Migrating cells were photographed under phase contrast microscopy. *Significantly different from control (*P *<* *0.01). The tested ruthenium complexes versus NAMI‐A group at the same dose, ^##^stands for *P *<* *0.01. (C–D) Effects of Ru on the invasion in 4T1 cells. Cells were pretreated with gemcitabine (1 ug/mL), NAMI‐A, G26b, and G94a (20 *μ*mol/L) treatment for 24 h. After 24 h, cells on the bottom side of the filter were counted. (1) control; (2) gemcitabine; (3) NAMI‐A; (4) G26b; (5) G94a. **Significantly different from control group (*P* < 0.01), and *P <* *0.05 compared with the control group.

Tumor cell migration and invasion are important steps in tumor metastasis, so we detected the effect of G26b and G94a on cell invasion and migration in vitro*,* using wound‐healing and Transwell assays. In the wound‐healing assay, the untreated HUVEC cells exhibited complete wound closure activity after treatment for 24 h. In contrary, the compound‐treated cells showed only a limited closure of wound at the end of their respective incubation time by forming asymmetrical protrusions into the denuded zone. G26b displayed most effective migration inhibition, comparing with NAMI‐A and G94a, which also inhibited cell migration significantly (Fig. 4A and B). The percentage of invasive inhibition ratio was 58–72% (20 *μ*mol/L). Consistently, the data from the Transwell assay indicated that invasion of 4T1 cells were obviously inhibited by RuCl4‐cynpy complexes (Fig. 4C and D).

### Network formation of HUVEC cells

**Figure 5:**
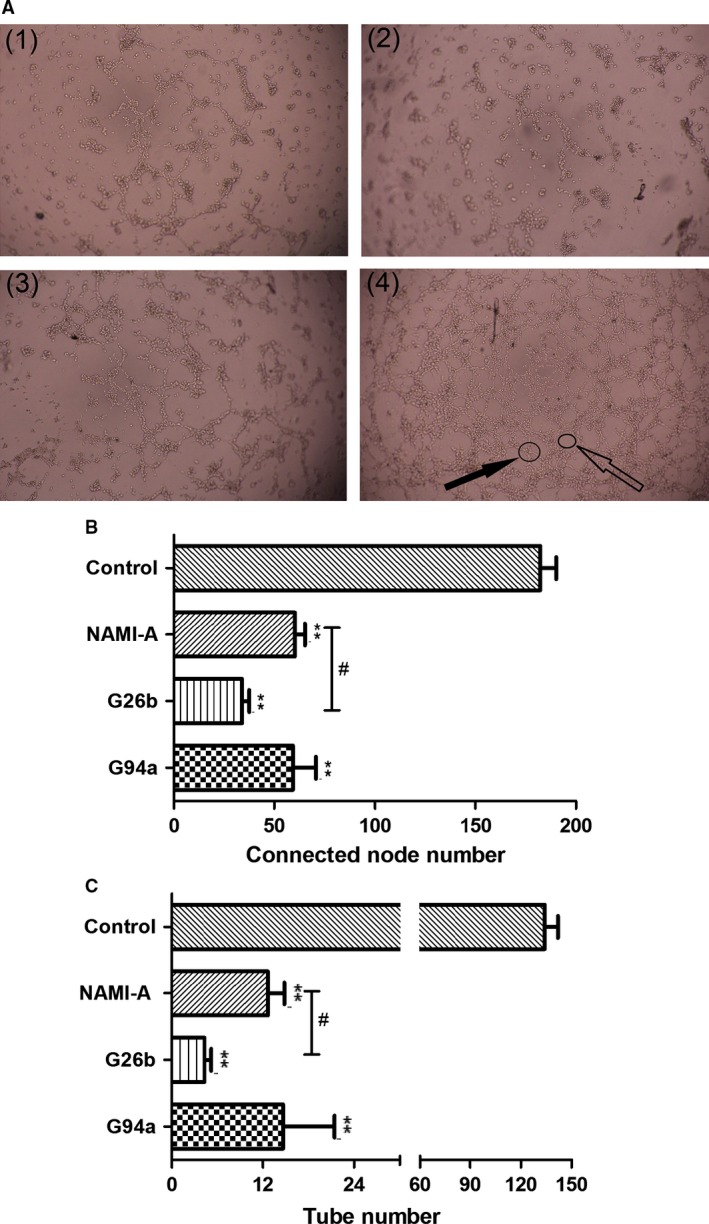
The effect of ruthenium complexes on human umbilical vein endothelial cells (HUVEC) tube formation. HUVECs were seeded on Matrigel‐coated 96‐well plates and incubated in the diluted medium containing 20 *μ*mol/L ruthenium complexes for 5 h at 37°C. (A) The images were captured under a phase contrast microscope at a magnification of 40× and observed the network‐like structures. (1) NAMI‐A; (2) G26b; (3) G94a; (4) control group. Solid black arrow stands for connected nodes, and feint arrow stands for tubes. (B) The connected node number of HUVEC; (C) The tube number of HUVEC. Values represent mean ± SD from three independent experiments. **P <* *0.01 compared with the control group. G26b versus NAMI‐A group at the same dose, ^#^stands for *P *<* *0.05.

The formation of tube‐like structures is an essential step in angiogenesis. This process involves matrix degradation, rearrangement, and apoptosis of endothelial cells 23. We then examined the effects of these complexes on the network formation of HUVEC cells. As revealed by Figure 5, increasing concentrations of RuCl4‐cynpy complexes significantly inhibited network formation of HUVEC cells. The effect on capillary‐like formations was evident in the number of network formation per field as well as on the branches of these tube‐like structures. In the control group, the capillary tube structures were observed on the basal membrane 5 h after HUVECs were placed in the wells. By contrast, the RuCl4‐cynpy complex treatment, especially G26b, significantly reduced the formation of tube‐like structures. Only a few tube‐like structures were formed in the cells treated with 20 *μ*mol/L G26b, whose inhibitory effect was stronger than that of NAMI‐A at the same dosage (Fig. 5).

### Inhibited levels of MMP‐2, MMP‐9, and VEGF in MCF‐7 cells

**Figure 6:**
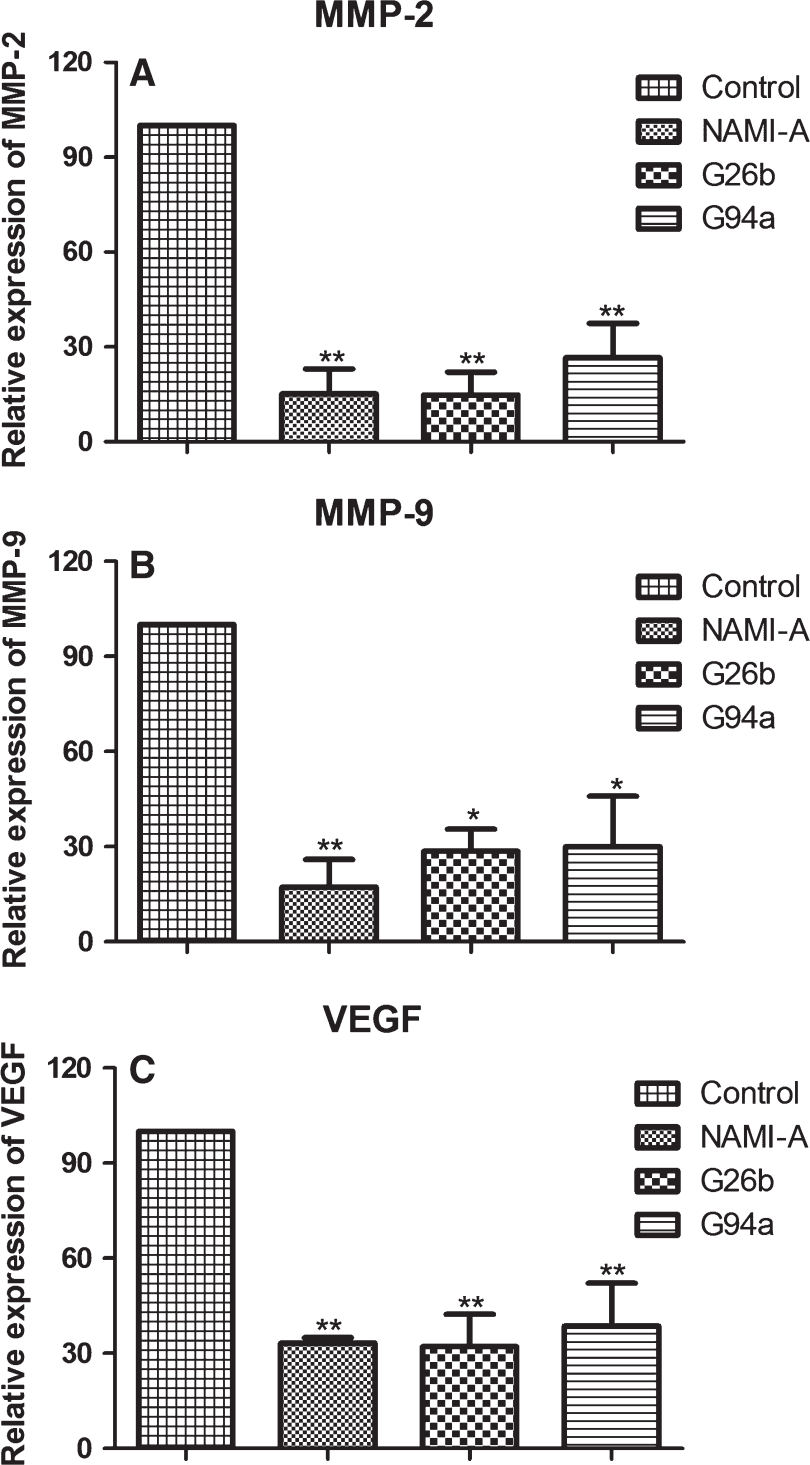
Representative RT‐PCR showing changes in the levels of associated gene level in migration and invasion of MCF‐7 cells. A total of 4 × 10^5^ cells were incubated in the diluted medium containing 20 *μ*mol/L ruthenium complexes for 24 h at 37°C. (A) Matrix metalloproteinase (MMP)‐2, (B) MMP‐9, and (C) VEGF expression in ruthenium (III) complex‐treated cells. Values represent mean ± SD from three independent experiments. *P <* *0.05 and **P <* *0.01 compared with the control group.

MMPs play an important role in ECM degradation for cancer growth, invasion, and cancer‐induced angiogenesis. In addition, VEGF is one of the central regulators of vessel development 24 and critical in angiogenesis and important for tumor metastasis 5, 24, 25. So we detected the transcription of metastasis‐related genes using real‐time PCR. Human mammary carcinoma MCF‐7 cells were treated with RuCl4‐cynpy complexes for 24 h. Gene transcription of MMP2, MMP9, and VEGF was inhibited significantly by treatment with ruthenium complexes (Fig. 6). However, no significant difference was detected among cells treated with these ruthenium complexes.

### Biodistribution of RuCl4‐cynpy complexes

**Figure 7:**
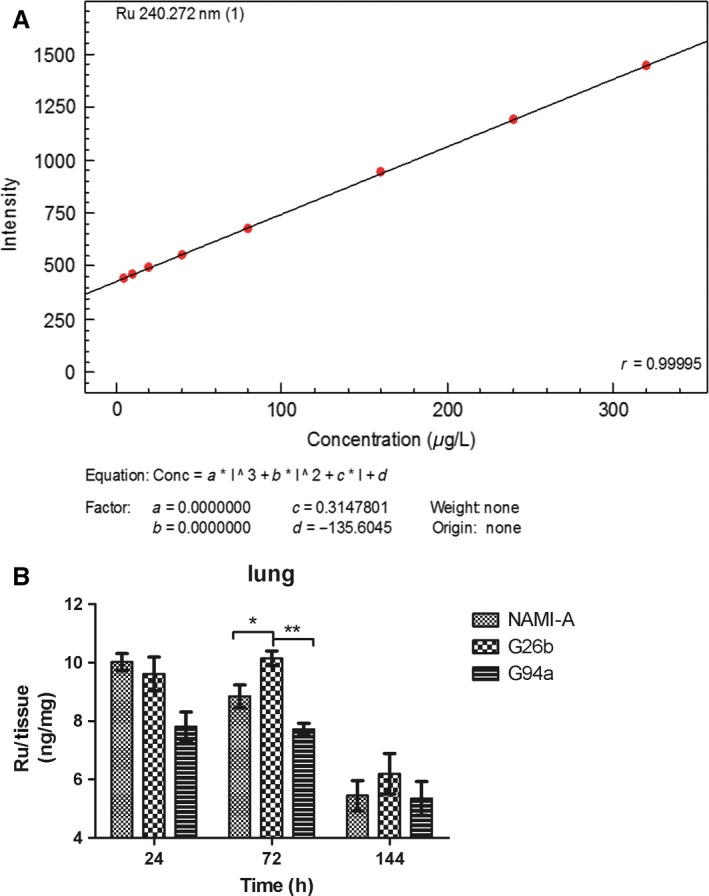
The elimination of ruthenium‐pyridine complexes from lungs of mice bearing 4T1 mammary carcinoma and treated with doses of NAMI‐A active on lung metastases (*n* = 3). The dosage regimen was 17.5 mg/kg per day for consecutive 6 days plus 3 days at 1‐day interval. The organs were collected at 24, 72, and 144 h after last administration. (A) The calibration plot of analyses and (B) the biodistribution of Ru in the lungs were detected.

Analysis of ruthenium uptake by lungs of cancer‐bearing mice treated with the tested ruthenium complexes were determined by induced coupled plasma spectroscopy. The ruthenium remained in the lung much longer and stayed at a higher concentration. Figure 7A was the standard curve. About 72 h after administration, ruthenium remained at a significantly higher concentration in the lungs for mice treated with G26b and the high ruthenium concentration (>6 ng Ru/mg tissue) lasted to 144 h (Fig. 7B). During the study period, the Ru was able to reach exceptionally high concentrations in the lung tissue of mice tested, helping explain the antimetastasis effects of G26b.

## Discussion

The study of metal‐based complexes as potential drugs in cancer chemotherapy is mainly based on the enormous knowledge derived from the clinical success of platinum analogs. Cisplatin quickly became the most widely used chemotherapeutic drug for the treatment of a wide range of malignancies since 1960s and clinical approval of platinum‐based derivatives, such as carboplatin and oxaliplatin, still remains commonly used today 26. Although platinum is extremely effective against several cancers, its application is also accompanied by severe side effects, such as nausea, cardiovascular abnormality, central nervous system symptoms, vomiting, and hearing loss, and many cancers show intrinsic or acquired resistance against the drug 27.

Ruthenium, as a platinum‐group metal, has also been found to display some favorable properties, and less toxicity than other transition metal compounds because of the potent similarity of ruthenium to essential metal irons 28. Ruthenium complexes have been widely investigated and two typical complexes, NAMI‐A and KP‐1019, are under clinical trials for metastatic and colorectal cancers 12. NAMI‐A showed negligible antiproliferative activity against cancer cells in vitro. However, the antimetastatic activity of NAMI‐A has been well studied 29. It is worthwhile to test if other classes of ruthenium compounds share any of these properties. The ruthenium complexes, G26b and G94a, were synthesized because ruthenium (III) was thought to be a more active and less toxic form of ruthenium.

Same to NAMI‐A, these two complexes did not induce significant cytotoxicity in vitro. We first detected the cell viability after G26b or G94a treatment. Little antiproliferative activity against many cancer cell lines and HUVEC (data not shown) was detected. Advantageously, they are supposed to have little host toxicity when applied in vivo 30.

Next, the in vivo anticancer activity of the RuCl4‐cynpy complexes and the reference compound NAMI‐A was tested in the mouse 4T1 mammary cancer model. When BALB/c mice are transplanted with 4T1 cells, metastasis spontaneously spreads to the lungs, but not to other tissues. This cancer growth and metastasis characteristics are very similar with breast cancer in human. In most cases, the lungs are the first organ that the detached cancer cells come upon most frequently and become the main location for cancer metastasis 31. We selected dosage on the basis of very promising anticancer activity results of reference compound NAMI‐A on model systems involving much lower toxicity of implantational cancer genesis. Taking into account characteristics of 4T1 cells, three times maintenance therapy was adopted after classic six consecutive treatments 32, 33. Through the in vivo model, we found that the absence of directly eradicate primary tumors in vivo was consistent with the results in vitro cell cytotoxicity, and our data were identical with those reported for NAMI‐A 22, 29. G26b and G94a could significantly inhibit pulmonary metastasis of mouse 4T1 breast cancer rather than primary tumors. The antimetastatic activity of G26b was significantly effective than that of NAMI‐A. Meanwhile, the biodistribution of RuCl4‐cynpy complexes in the lungs was determined. Ru was kept in a relative higher concentration and remained longer in the lungs in G26b group than G94a and NAMI‐A groups, which may be a key factor for the better antimetastasis activity of G26b, because the longer retention time gives it prolonged contact with both the new vessels and metastatic cells in the lungs. According to the biodistribution experimental results, it was assumed that the metabolic stability of G26b was enhanced, compared with that of NAMI‐A and G94a.

Ineffectiveness of currently available cancer treatments is mainly due to the invasive and metastatic properties of malignant cancer cells. The invasive, metastatic, and angiogenesis in cancer involves matrix degradation, rearrangement, and apoptosis of endothelial cells 23. Therefore, this cellular process provides a multiple targets for the development of therapeutic agents to inhibit cancer invasion and metastasis 1, such as MMP‐2, ‐9, and VEGF. In this report, we examined antitumor and antimetastasis effects of RuCl4‐cynpy complexes and investigated their probable mechanism. In vitro invasion and migration assays, including Transwell and wound‐healing assays were used to investigate the inhibitory effects of RuCl4‐cynpy complexes on the invasive potency. The data obtained from the Matrigel invasion assay showed that RuCl4‐cynpy complexes at 20 *μ*mol/L inhibited the invasion of 4T1 cells, by over 60% (Fig. 4C and D). Similarly, the data from the wound‐healing assay indicated that migration of HUVEC was inhibited by G26b and G94a, and not due to their direct cytotoxicity.

The formation of tube‐like structures is an essential step in angiogenesis. We then examined the effects of RuCl4‐cynpy complexes treated HUVEC cells in each of metastasis phases. Beside in antiinvasion and antimigration, G26b also significantly reduced the formation of tube‐like structures more significantly than NAMI‐A did. Meanwhile, it is found that angiogenesis‐related VEGF was also down‐regulated. In our studies, G26b displayed prominence than G94a in antiinvasion and antimigration.

Notably, the antimetastatic activities of G26b was more significant than NAMI‐A**.** Beside its inhibitory effect on angiogenesis, it has been proved that metastatic cells were targets for NAMI‐A in preference to primary cancer cells 22. Ru remained longer time in the lungs, may be a key factor in the antimetastatic activity of G26b. Because longer remain time gave it prolonged contact with the metastatic cells in the lungs.

Here, we also confirmed ruthenium drugs execute their anticancer activity by acting on cancer metastasis. And RuCl4‐cynpy complexes were assumed to exhibit a clearer selective toxicity for lung metastases than for primary cancer or other healthy “normal” tissues.

## Conclusions

Novel RuCl4‐cynpy complexes, G26b and G94a, displayed effective antimetastatic effect in vitro and in vivo. The longer Ru‐elimination time in the lungs may be one reason for the better antimetastatic effect, compared with NAMI‐A. These two ruthenium (III) complexes are promising to develop as antimetastatic reagents.

## Conflict of Interest

The authors declare no conflict of interest.
